# Hermetic storage of okra seed maintains seed longevity under changing environment

**DOI:** 10.1371/journal.pone.0287476

**Published:** 2023-06-15

**Authors:** Muhammad Amir Bakhtavar, Irfan Afzal, Ehsan Khalid, Nazish Jabeen, Raheela Jabeen

**Affiliations:** 1 Institute of Plant Breeding and Biotechnology, Muhammad Nawaz Shareef University of Agriculture, Multan, Pakistan; 2 Seed Physiology Lab, Department of Agronomy, University of Agriculture Faisalabad, Faisalabad, Pakistan; 3 Institute of Horticultural Sciences, University of Agriculture Faisalabad, Faisalabad, Pakistan; 4 Department of Biochemistry and Biotechnology, The Women University Multan, Multan, Pakistan; Manonmaniam Sundaranar University, INDIA

## Abstract

Okra seed is vulnerable to loss of germination and vigor in variable storage conditions. High seed moisture contents (SMC) accelerate seed deterioration during storage thus keeping low seed moisture contents by storing seed in hermetic bags may help to retain seed longevity. Okra seed was equilibrated to four initial moisture levels including 8,10, 12 and 14% SMC. Seed was then packed and stored in traditional storage bags (Paper, cloth, polypropylene and jute bag) and hermetic Super Bag for 12 months under ambient conditions. Seed stored in hermetic Super Bag at 8 and 10% moisture contents maintained higher germination due to low seed moisture contents. Moreover, activities of α-amylases and total soluble sugars were higher while electrical conductivity of seed leachates, malondialdehyde (MDA) and reducing sugar contents were less in the seeds stored in hermetic Super Bag at 8 and 10% SMC as compared to seed stored in traditional storage bags. Hermetic storage at 14% moisture negatively influenced the seed quality. Moisture adsorption isotherms of okra seeds were developed at constant temperature of 25°C and varying levels of relative humidity from 60 to 90%. Moisture isotherms indicated no significant increase in seed moisture contents at 60 and 70% relative humidity (RH) in hermetic bags whereas a minor increase in seed moisture at 80 and 90% RH has been observed for the seeds incubated in hermetic bags. SMC significantly increased in traditional storage bags particularly in jute bag at high RH. In conclusion, storage in hermetic bags, maintain low seed moisture and high seed quality. Okra seed storage in hermetic bags at 8 and 10% SMC maintains seed longevity under ambient storage conditions.

## Introduction

Okra (*Abelmoschus esculentus* L.) is an important vegetable, cultivated across the globe on an area of 2531 thousand ha during 2020 with total production of 10548 thousand tons [1]. Throughout the world, farmers and growers demand for quality seeds with high genetic purity, germination and vigor to achieve uniform and successful establishment of a weed-free crop [2]. Pakistan is mainly importing vegetable seed and major reason for this huge import is poor quality of local seed which is linked to improper storage practices. Due to lack of access to certified seeds, farmers have to use carryover seed which is not of good quality resulting into poor and uneven crop stand and ultimately low yield [3].

**Fig 1:**
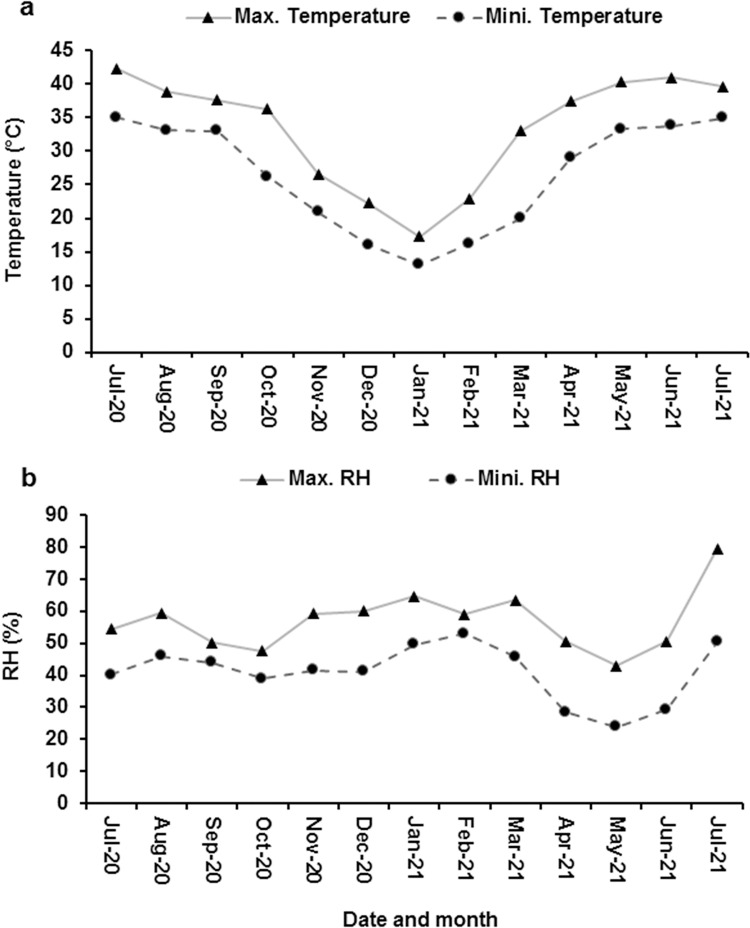
Maximum and minimum temperature (a) and relative humidity (b) recorded from storeroom on monthly basis.

Okra seed contain around 15% oil on dry weight basis [4]. Seeds having higher oil contents deteriorate faster due to lipid peroxidation and oxidative stress [5, 6]. Water present in oily seeds is mainly thought to be free water due to low affinity of fats for water [7]. Fluctuating RH and temperature of the store room have impact on seed moisture contents and ultimately its quality [8]. In Asia particularly Pakistan, most of the rainfall is received in moon soon season (July to September), which results into higher RH during these months [9]. In Pakistan, okra is planted during the month of February [10] and seed is harvested in June and then stored for sowing in next season. So, there is a need to need to develop low cost on farm seed storage technique that could be easily implemented by majority of farmers. Okra seed storage period coincide with months having high temperature and rainfall (Fig 1A and 1B). In such conditions seeds are equilibrated to higher MC, and amount of free water increased which enhance the rate of reactions involved in seed deterioration [11]. Moisture isotherms describes the relationship between ambient relative humidity and seed moisture contents at given temperature [12]. Adsorption isotherms are used to estimate the changes occurring in the stability of biological materials [13].

Seed moisture contents, temperature of the storage environment and length of the storage period are the most important factors, that can affect the seed storage life of seed [14]. Increased seed moisture is the main culprit responsible for seed deterioration and reduce seed germination [15]. Seed viability can be retained for significantly long period if seeds are stored under low temperature and moisture conditions. Storage life of orthodox seeds under ambient conditions will be short while seed stored under low temperature (-18-20°C) and low humidity will have longer storage life in a rage of 40–60 years [16]. These low temperature conditions are generally maintained for Genebank storage. The ideal approach for common farmers is to dry the seed properly and then control seed moisture through proper packaging. Vapor transmission rate of different packaging materials vary according to their mesh size [17] and similarly moisture contents of the seed stored in these packaging materials may vary. Large mesh size of traditional storage bags including cloth, jute and polypropylene bags offer no hindrance to the incoming water vapors under high ambient RH conditions and these vapors are quickly absorbed by the seeds [18]. Seed storage in hermetic bags maintain low seed moisture and have been reported to prolong the storage life of stored maize and wheat seed [15]. Hermetic bags provide cost effective and organic seed storage solution for wheat without any requirement of fumigation [19]. Hermetic Super Bags of GrainPro Inc. USA have been made from three layers of high strength polyethylene sandwiched to make a single layer. In each layer of polyethylene there is a gas coating which serve as barrier layer to limit vapors and oxygen ingress.

Only a few multinational seed companies in developing countries adopt cold storage facility to store their seeds while rest all of the local seed companies and farmers (82.7%) store their seeds in traditional way without controlling the temperature and relative humidity of the store. Majority of the farmers cultivate open pollinated cultivar Sabz Pari and make its seed that is stored and used for sowing in next season. But the main problem faced by farmers is lack of proper storage facility. Cold storage is uneconomical and unaffordable by small-scale farmers due to frequent energy and infrastructure requirement. Hermetic storage in Super Bag is a viable and economical option for seasonal storage of seeds at ambient conditions. This storage technique form the basis of Dry Chain technology that rely on initial drying to low seed moisture contents and maintenance of that low moisture during storage and supply chain through hermetic packaging [20]. Considering high seed moisture as main culprit of reduced seed viability and vigor, prospects of hermetic storage in Super Bag as an alternative and economical seed storage technique has been explored in this study. The quality of stored seed was also compared in traditional storage bags at various levels of moisture contents.

## Materials and methods

### Experiment and treatment details

The seed of commercial cultivar of okra named Sabz Pari was obtained from Ayub Agricultural Research Institute Faisalabad for research purpose through official correspondence. Initial seed germination was 99.5 while seed moisture contents were 10%. Okra seed (5 kg) was equilibrated to various moisture levels i.e. 8, 10, 12 and 14%. Seed was then packed in traditional storage bags (Paper, cloth, polypropylene and jute bag) and hermetic Super Bag having total capacity of 10 kg. Super Bags were closed manually by pressing the bag from open end to remove as much as air as possible and then open end was twisted, folded back and tied with tie wrap. Storage bags were placed at storeroom of Seed Science and Technology Department, University of Agriculture Faisalabad, Pakistan for 12 months. Bags were opened after six months for collecting seed samples and then closed again adopting the same procedure and placed in storeroom for further storage. After each sampling, seed moisture contents, germination and biochemical attributes were determined.

### Data of maximum and minimum temperature and relative humidity of storeroom

Relative humidity and temperature of the storeroom from July 2020 to July 2021 was recorded with humidity and temperature DataLoggers® (Fig 1A and 1B). Relative humidity was maximum 79.5% during the month of July 2021. Lowest relative humidity (23.8%) was observed during the month of May 2021. Temperature was maximum (42.3°C) during July 2020 while minimum temperature (13°C) was recorded during January 2021.

### Seed drying and equilibrating seed moisture contents

Seed drying was done in airtight boxes by mixing okra seed with seed drying beads. Drying beads are made up of zeolites and contain microscopic pores that can absorb and tightly hold the water molecules. A beads calculator [20] was used to calculate the amount of beads required for drying seed to required moisture levels. To lower down the moisture contents of 5 kg okra seeds from 10 to 8%, a total of 621 g drying beads were used at drying temperature of 30°C. As initial seed moisture contents were 10% so seeds were packed as such at this moisture level. Equation based on the oven dry method of International Seed Testing Association (ISTA) was used to quantify the required volume of water to equilibrate the seed at 12 and 14% moisture contents [21].


$$Amount\;of\;water\;required=\left[(\frac{100-Initial\;SMC}{100-Final\;SMC}\times Seed\;weight)-Seed\;weight\right]$$ (1)

In order to increase seed moisture from 10% to 12%, a total 114 ml water was sprayed on 5 kg okra seed with continuous mixing in airtight jar. Similarly, 233 ml water was sprayer and thoroughly mixed with 5 kg okra seed to change the moisture from 10 to 14%. After 24 hours incubation in airtight jars, the desired level of seed moisture contents were achieved.

### Determination of seed moisture contents

Seed moisture contents on fresh weight basis were determined by low-constant-temperature oven method as per Chapter 9 of International Rules for Seed Testing given by International Seed Testing Association (ISTA). Seed sample (5 g) was dried at 103°C for 17 hours and dry weight was recorded [21]. Seed moisture contents was calculated by using Eq 2.


$$Seed\;moisture\;contents\;(\%)=\frac{Fresh\;weight-Dry\;Weight}{Fresh\;weight}\times 100$$ (2)

### Seed germination testing

Seed germination was tested by placing 100 seeds in sterilized and well moist blotting paper. Seed samples were randomly drawn from each bag after 6 and 12 month storage. From each sample, germination test was conducted by using 400 seeds in the form of 4 replications as per instructions given in Chapter 5 of International Rules for Seed Testing [21]. The germination test was conducted at alternating temperature of 20 and 30°C with lower temperature for 16 hours and higher for 8 hours.

### Seed vigor test

Accelerated aging treatment was given to the seeds by incubating it in a growth chamber (F.lli Della Marca S.r.l. Rome) already set at 45°C temperature and 95% relative humidity. Duration of the incubation period was 72 hours [22]. After aging treatment, germination of seed samples was determined by following ISTA rules for seed testing as given in previous section.

### Determination of seed leachates EC

From each bag, seed samples were drawn of which 50 seeds were counted and their weight was recorded. These 50 seeds were then soaked in a beaker having 250 ml distilled water. Electrical conductivity of the distilled water should be less than 5 μS cm^-1^. After seed soaking, the beakers were covered with aluminum foil and then placed at 20 ± 2°C for 24 hours [21]. After 24 hours soaking, seed and solution was stirred gently. Electrical conductivity of the solution was measured by using an electrical conductivity meter (HI 99300).

Following equation was used to calculate EC in μS cm^-1^ g^-1^.


$$Conductivity=\frac{Conductivity\;reading-Background\;reading}{Weight\;of\;replicate\;(g)}\times 100$$ (3)

### Seed biochemical attributes

The activity of α-amylase was measured in 0.5 mg seed sample extracted in 5 mL sodium phosphate buffer [23]. The reducing sugars were measured by DNS method [24].

Total soluble sugars were by following the Anthrone method [24]. Malondialdehyde contents (MDA) were quantified from the 0.5 g seed uniformly ground in 10% Trichloroacetic acid [25]. Absorbance was taken at three different wavelengths i.e. 450, 600 and 532 nm. Following Eq (4) was used to calculate MDA.


$$MDA\;(nmol/g\;FW\;or\;DW)=6.45\;(A_{532}-A_{600})-0.56\times A_{540}$$ (4)

### Development of seed moisture isotherm

In a separate experiment, 200 g okra seed was filled in small size hermetic Super Bag and traditional storage bags (paper, poly propylene, cloth and jute bag). For each level of RH separate set of Super Bag and all traditional bags was prepared. These bags containing seeds were then incubated at 60, 70, 80 and 90% RH and constant temperature of 25°C. After 15 days incubation period, bags were opened and seed moisture contents were determined as described in methodology of previous experiment. Selected levels of RH were maintained in growth chamber. Moisture adsorption isotherms were drawn in Microsoft Excel by keeping different levels of RH at x-axis and data of seed moisture contents at y-axis.

### Experimental design and statistical analysis

Experiment was conducted by using Completely Randomized Design (CRD) with factorial arrangement. Experiment was replicated thrice. Line graphs has been made from mean values and standard error bars have been added. P values were mentioned to show the significance levels. Biplots were made by using R statistical computing software.

## Results

### Seed moisture contents

**Fig 2:**
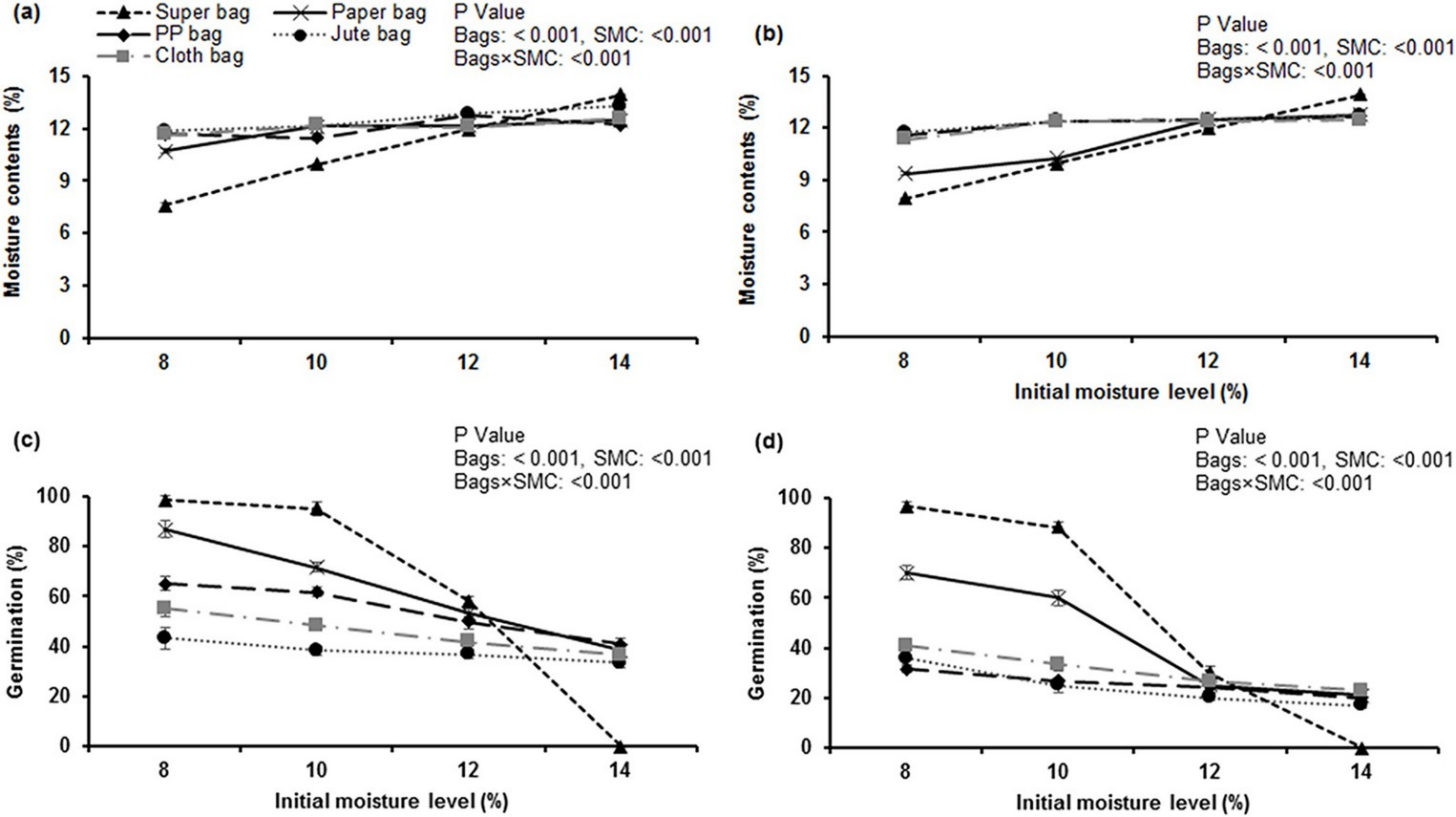
Impact of initial moisture levels and storage bags on final seed moisture contents and germination of okra seeds after 6 and 12 months of storage. Initial germination 99.5%.

After storage period of 6 months, seed stored in hermetic bag at 8% had significantly (P ≤ 0.05) lower SMC. Seed stored at 14% moisture contents in hermetic bag had highest seed moisture contents (Fig 2A). Overall, hermetic storage almost maintained initial moisture levels whereas seed moisture increased in traditional storage bags where seeds were stored at 8 and 10% moisture contents. While a decrease in moisture contents was observed where seeds were stored in traditional storage bags at 14% moisture contents during the period when ambient RH was low. Similarly, after 12 months storage period, highest SMC were given by the seeds stored in hermetic bag at 14% moisture contents. Okra seeds in hermetic bags at 8% SMC maintained almost initial moisture (7.59%) levels (Fig 2B).

### Germination

Okra seed stored in hermetic bags at 8% seed moisture contents gave highest germination (98.33%) after 6 months and it was statistically similar to the germination of seed in hermetic bags at 10% moisture contents. However, seeds at 14% moisture contents in hermetic bag experienced a complete loss of germination within 6 months (Fig 2C). Germination decreased in traditional storage bags especially in jute bag (43.33%) within first six months. After 12 months storage, germination of seeds stored in hermetic bags at 14% initial was reported zero as these seeds have already lost germination within first six months (Fig 2D). A significant decline (almost 70%) in germination of okra seeds was measured in traditional storage bags at all levels of initial seed moisture. Similarly, germination of the seed in hermetic bag at 12% moisture contents also dropped (69.5% reduction) significantly after storage period of 12 months.

### Seed vigor

**Fig 3:**
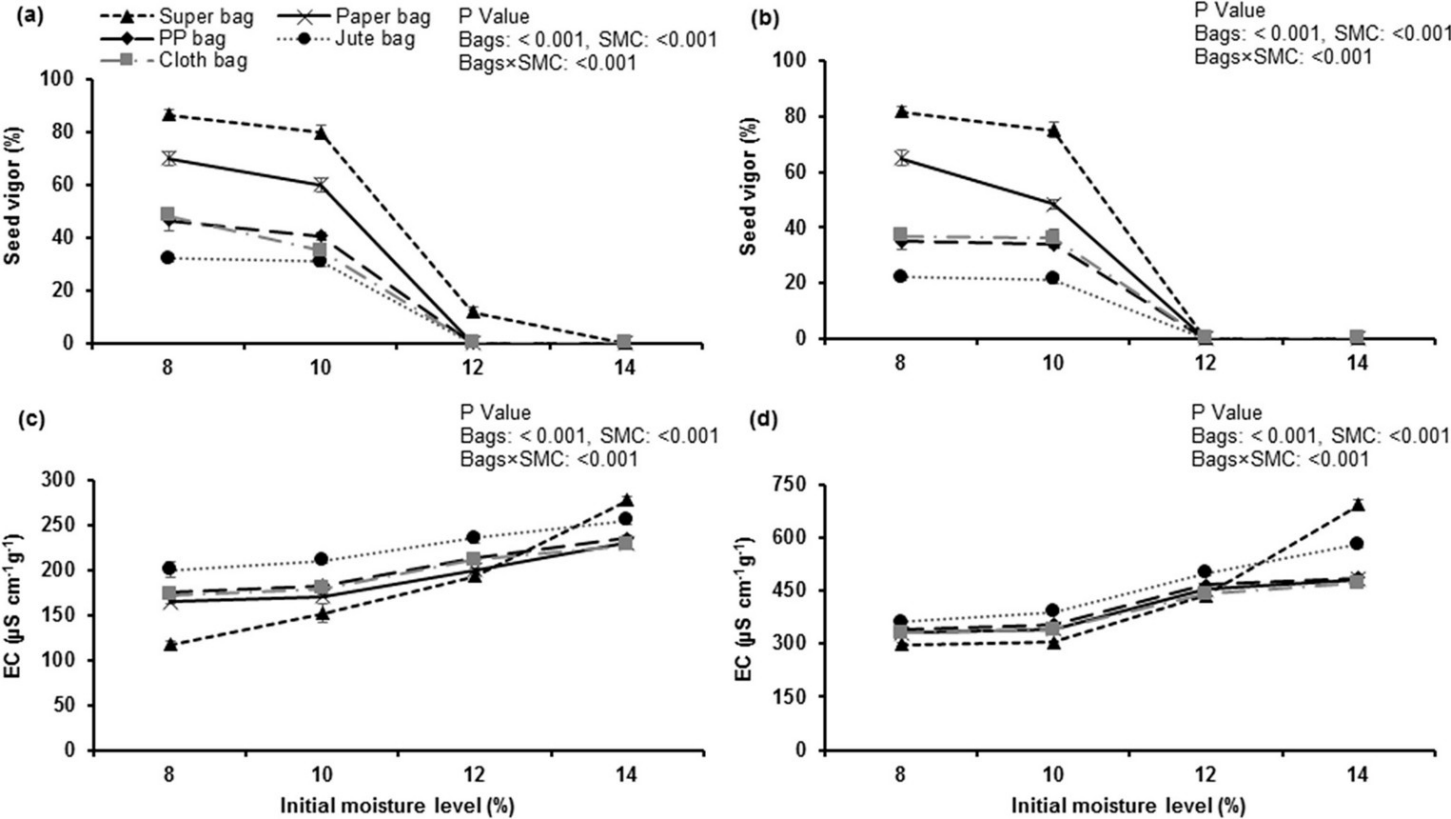
Impact of initial moisture levels and storage bags on seed vigor and electrical conductivity of okra seed’s leachates after 6 and 12 months of storage.

After 6 and 12 months of storage, vigor (germination following accelerated aging) of seed stored in different bags at 8, 10, 12 and 14% moisture contents varied significantly. After storage period of 6 months, okra seeds in hermetic bag at 8% moisture contents gave 86.67% vigor and it was statistically at par to the vigor of seed in hermetic bag at 10% moisture contents. Okra seed in hermetic bag and traditional storage bags at 14% moisture contents completely lost its vigor (Fig 3A) within first 6 months. After 12 months, vigor was higher (81.67%) for the seed stored in hermetic bag at 8 and 10% moisture contents. Seed storage in traditional storage bags and in hermetic bag at 12 and 14% moisture contents resulted in complete loss of vigor (Fig 3B). Overall seed storage in hermetic bag (at 8 and 10% SMC) maintained higher seed vigor while seed in traditional storage bags lost its vigor quickly.

### Seed’s leachates electrical conductivity

Electrical conductivity (EC) of okra seed leachates after 6 months of storage was markedly less for the seed in hermetic bag at 8% moisture contents. Seed’s leachates EC was maximum for hermetically stored seed at 14% moisture contents (Fig 3C). Similarly, EC of seed leachates after 12 months was significantly higher for hermetically stored seed at 14% moisture contents. Seed leachate’s EC was the lowest when stored in hermetic bag at 8 and 10% moisture contents (Fig 3D). EC of seed’s leachates also increased in traditional storage bags at all levels of seed moisture contents, especially in jute bag.

### Biochemical attributes

**Fig 4:**
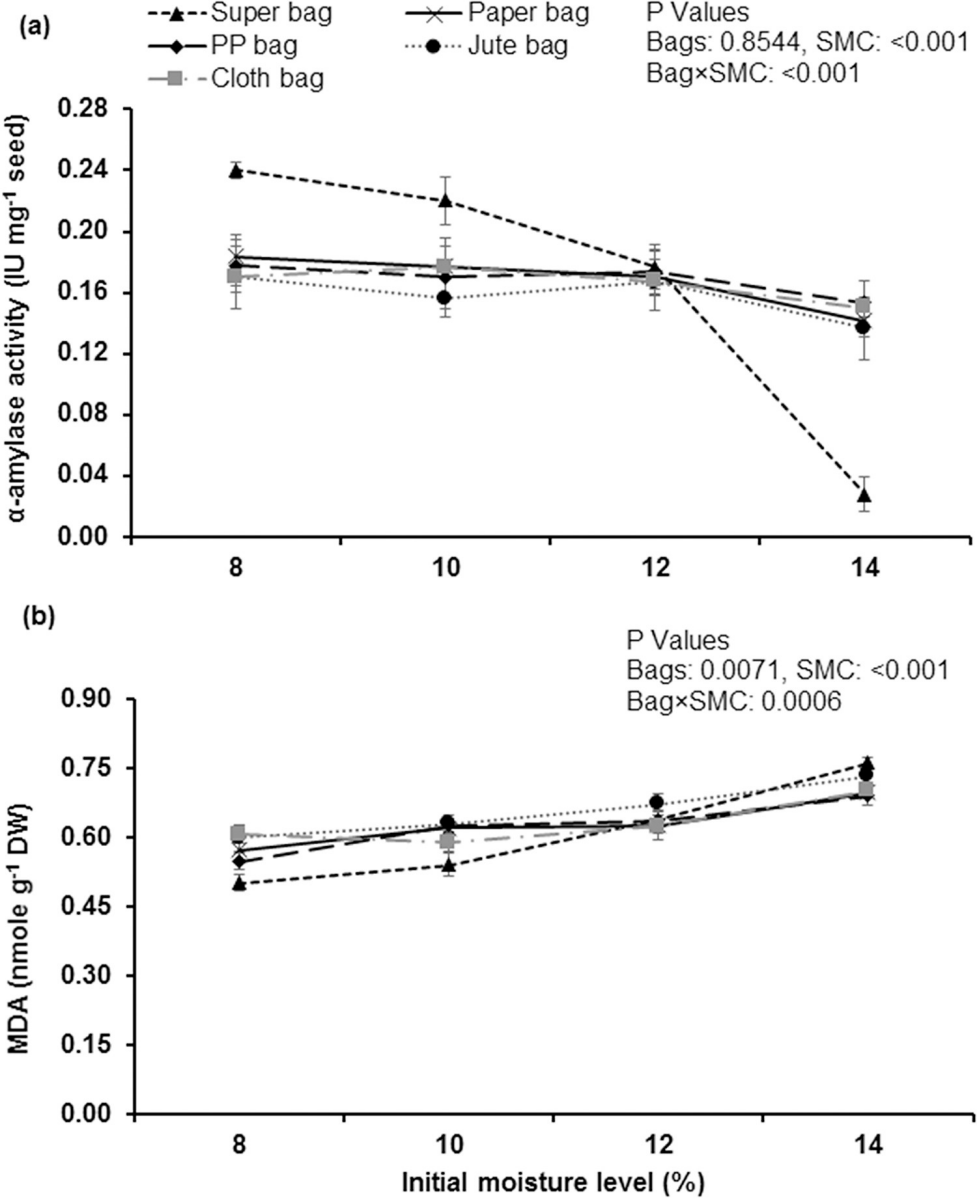
Impact of initial moisture levels and storage bags on activities of α-amylases (a) and MDA contents (b) of okra seed stored for 12 months.

Maximum α-amylase activity was measured in seeds stored in hermetic bag at 8 and 10% moisture contents. Seeds stored in hermetic bag at 14% moisture contents showed minimum α-amylase activity (Fig 4A). Lowest malondialdehyde contents were recorded from the seed that were stored in hermetic bags at 8 and 10% SMC. Whereas, seed stored in hermetic bag and in jute bag at 14% moisture had maximum MDA contents (Fig 4B). Similarly, α-amylases activities decreased while MDA contents increased in traditional storage bags at all levels of initial seed moisture contents.

**Fig 5:**
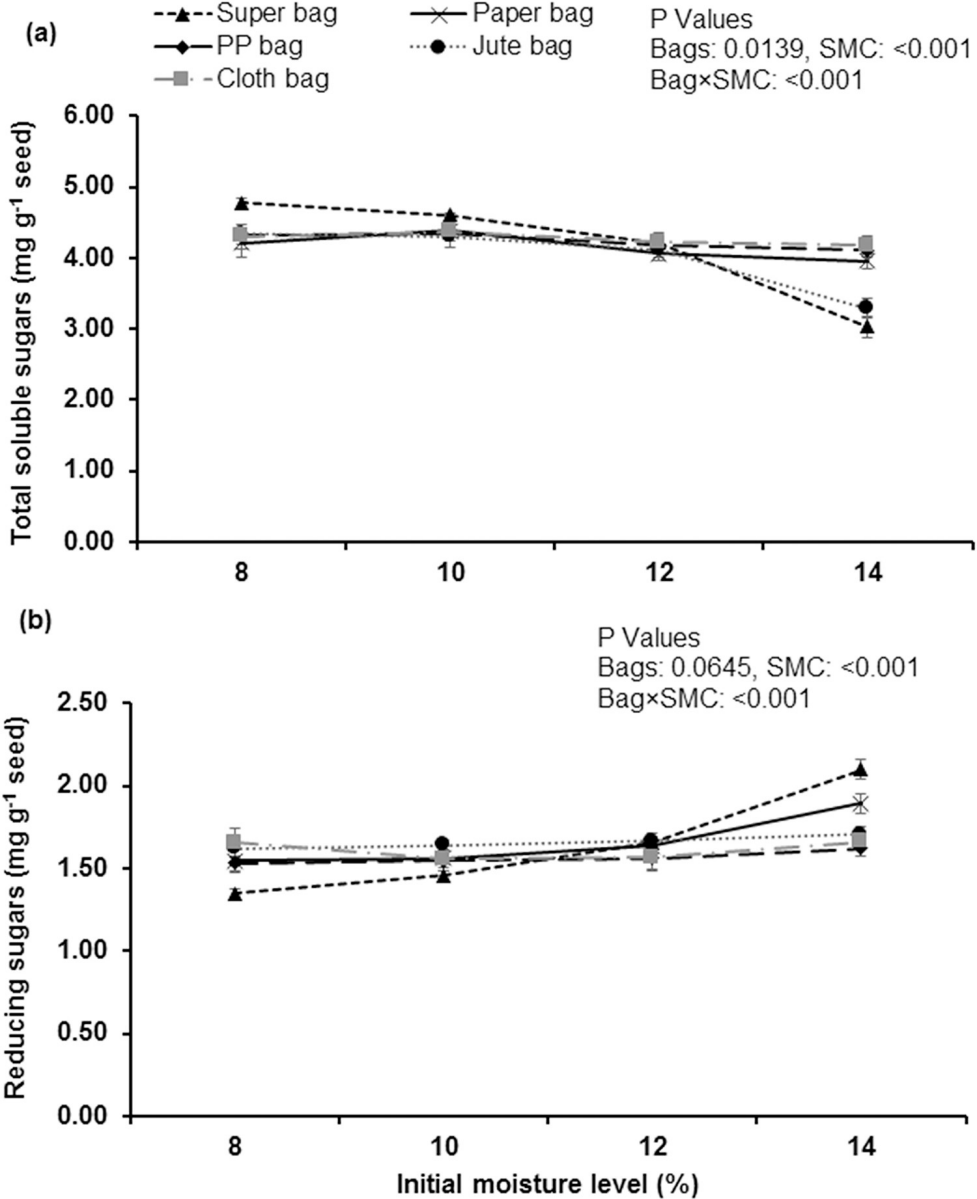
Impact of initial moisture levels and storage bags on total soluble sugars (a) and reducing sugars (b) of okra seed stored for 12 months.

Seed stored at 8 and 10% moisture contents in hermetic bag gave maximum values of total soluble sugar contents. Minimum total soluble sugar contents were measure in the seed that were stored at 14% moisture contents in hermetic bag and jute bag (Fig 5A). Maximum reducing sugars were quantified from the seeds stored at 14% SMC in hermetic bag followed by paper bag at 14% SMC. Minimum reducing sugars were measured from the seeds stored at 8 and 10% SMC in hermetic bag (Fig 5B).

### Correlation of seed moisture contents, germination and seed biochemical parameter

**Fig 6:**
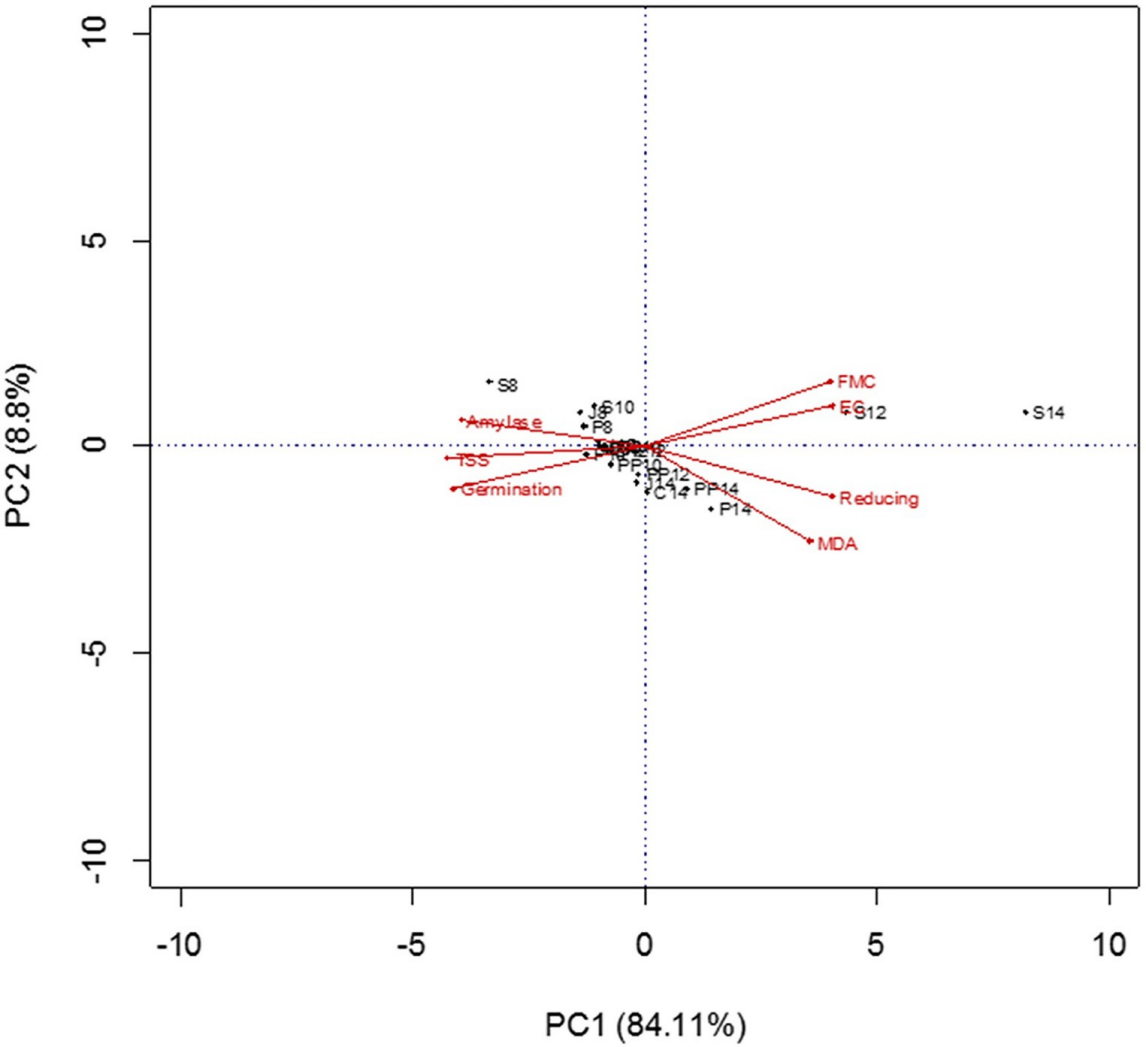
Biplot of seed germination, final moisture contents (FMC), electrical conductivity of seed leachates (EC), reducing sugars, total soluble sugars (TSS) and activities of α-amylases of okra seeds in various storage bags at different initial moisture levels. C; Cloth Bag, J; Jute Bag; PP; Polypropylene Bag, P; Paper Bag; S; Super Bag. Initial seed moisture contents: 8, 10, 12 and 14%.

Biplot of the seed moisture contents, germination and biochemical attributes showed that there was a negative correlation between final moisture contents and seed germination, activities of α-amylases and total soluble sugar contents. Final moisture contents were positively correlated electrical conductivity of seed leachates, malondialdehyde and reducing sugar contents (Fig 6). Electrical conductivity of seed leachates, reducing sugars and MDA contents increased while seed germination, activities of α-amylases total soluble sugar contents decreased in traditional storage bags and hermetic bags at high (12,14%) initial seed moisture contents.

### Moisture adsorption isotherm of okra seed

**Fig 7:**
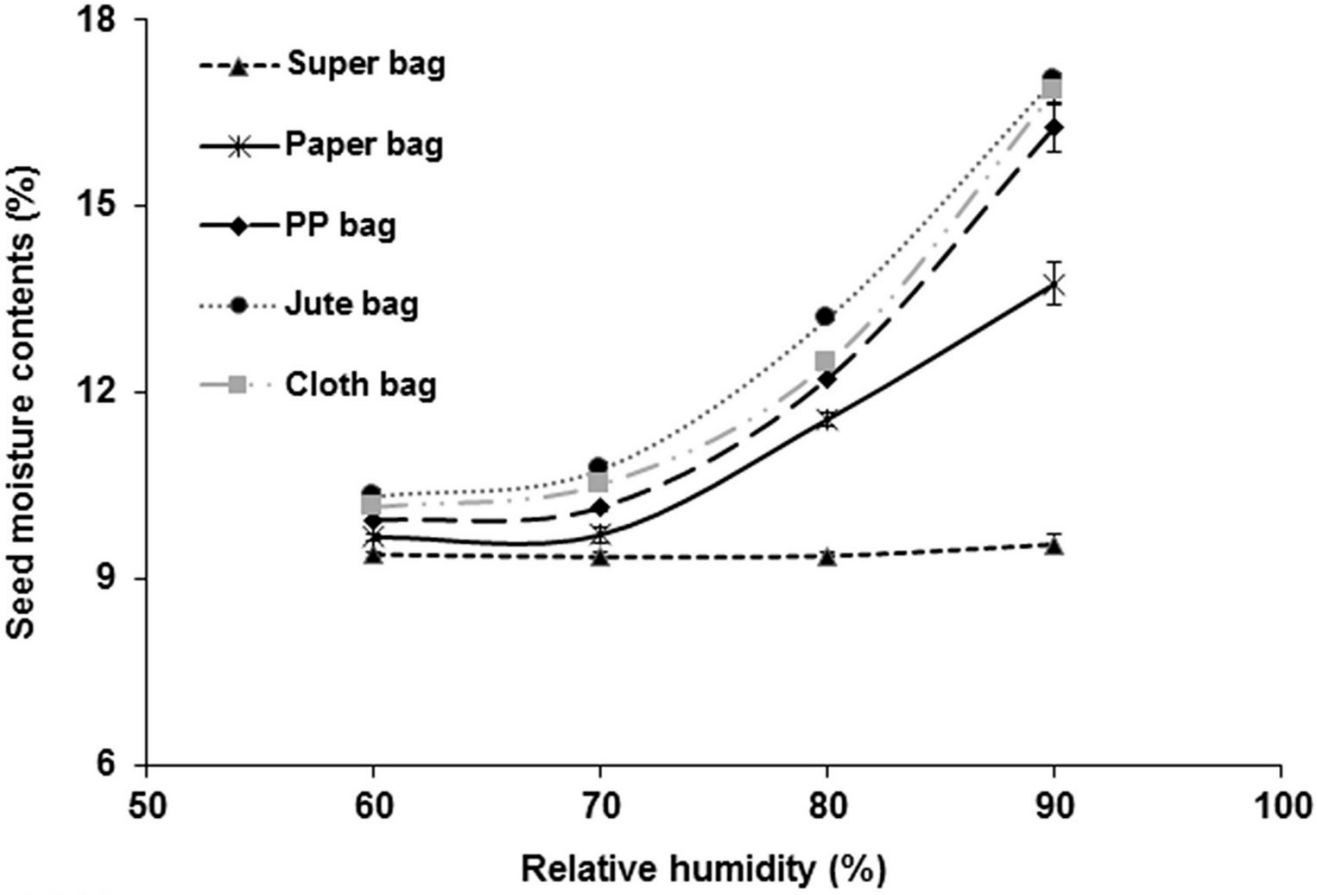
Moisture isotherms of okra seed in various at 25°C.

Okra seed in hermetic bag gave almost straight line moisture adsorption isotherm at various levels of RH. Moisture adsorption isotherms of traditional storage bags indicate that okra seed moisture increased with increasing RH (Fig 7). Highest moisture contents was recorded from seed in jute bag whereas seed placed in hermetic bag had low moisture at 60, 70 and 80% RH. At 90% RH, a huge difference in moisture contents of seed packed in traditional storage bags and hermetic bag was observed. Seed in hermetic bag had 9.36% moisture compared to jute bag (17.1%), cloth bag (16.86%), PP bag (16.28%) and paper bag (13.76%).

## Discussion

Moisture contents of okra (Fig 2A and 2B) seed were lowest when stored in hermetic bag at 8% moisture contents and higher (6% more) when stored in hermetic bag at 14% moisture contents. Seed can absorb and lose moisture due to its hygroscopic nature and come in equilibrium with the ambient humidity of surrounding air [26]. If there is any change in relative humidity and temperature of ambient air, it will influence the seed moisture contents and ultimately seed quality. Hermetic Super Bags restricted the free entry and exit of water vapors into seeds as water vapor transmission rate of hermetic bag is very low (≤5 gm^-2^ day^-1^) [27]. Moisture gain in traditional bags was the result of high RH prevailing in the storage environment (Fig 1B).

Nearly a straight-line moisture adsorption isotherm of okra seeds in hermetic bag clearly indicates that hermetic bag showed resistance to any significant change in seed moisture contents especially at high ambient RH. Contrary to this, moisture contents increased in traditional storage bags at higher RH levels. At 90% RH, seed stored in traditional jute bag had highest moisture (≈7.4% higher compared to hermetic bag). Traditional storage bags gain moisture under condition of high ambient RH. Hermetic Super Bag showed resistance to moisture increase whereas moisture contents of seeds stored in traditional woven PVC bags increased up to 4.2% [28].

As the storage period progressed, almost 70% reduction in germination of okra seed was observed. Minor reduction in germination of hermetically stored okra seed was observed at 8 and 10% SMC. Higher germination is the result of low seed moisture contents maintained in hermetic bag at 8 and 10% initial seed moisture contents throughout the study period. According to Harrington [29], seed storage life can be doubled by 1% reduction in seed moisture contents [29]. Data of store house indicated that maximum relative humidity was above 50% during most of the storage period. Similarly, maximum temperature was around 35°C during most of the storage period except the period from November 2020 to February 2021. These storage conditions are not ideal for okra seed especially in traditional storage bags as seed moisture will increase when ambient RH will increase as found in our study (Fig 2A and 2B). For ideal seed storage, sum of relative humidity (%) and storage temperature in degree Celsius (°C) should be less than 60 [29]. This was the possible reason for decline in okra seed germination in traditional storage bags. Seed germination drastically reduced in hermetically stored okra seed at 12 and 14% moisture contents (Fig 2D). Storage in hermetic bags at high moisture contents (14% SMC) is not adequate and resulted in seed deterioration. In hermetic bags, seeds were unable to lose moisture even when environmental RH was low as hermetic bag has negligible water vapor transmission rate. Reduction in germination of the seeds having high moisture in hermetic bags (14%) can be attributed to fast seed deterioration and aging process due to production of free radicals and reactive oxygen species [6]. Lipid peroxidation and oxidation of biomolecules have been considered as the major reason of seed deterioration during the aging process [5]. Seed with higher moisture had a higher water activity that accelerated the rate of lipid peroxidation and Maillard reaction [30, 31] thus seed germination is reduced. In hermetic system, seed MC must be lower (2–3%) than that at which seeds are normally packed in non sealed storage. Seed respiration rate is accelerated at high MC [32]. Food reserves being respired lead to losses in the vigour of stored seeds.

Presence of free oxygen in porous packaging materials favored the oxidative and seed deteriorative processes while hermetic bag safeguarded the seed viability by restricting the oxygen entry (≤4 ccm^-2^day^-1^).

The first effect of seed aging appeared to be reduced vigor followed by viability losses. Seed viability and vigor losses are highly dependent upon the storage temperature and seed moisture contents [5]. There is an inverse relationship between seed vigor and seed moisture contents [33]. High moisture contents of the seed stored in hermetic bags at 14% moisture contents and moisture gain in traditional storage bags due to high RH (Fig 1A and 1B) reduced the vigor of seeds. At high seed moisture contents, speed of Maillard reaction and rate of sugar hydrolysis also increased which is responsible for the reduced seed vigor [34].

Lipid peroxidation of biomolecules has major role in seed deterioration and aging process and as a result malondialdehydes (MDA) are produced. Maximum MDA contents were measured in okra (Fig 4B) seed stored in traditional and hermetic storage bags at 14% moisture contents. High seed moisture contents resulted into higher activities of reactive oxygen species (ROS) in the seeds that resulted into production of MDA through lipid peroxidation [6]. Free radicals produced by lipid peroxidation and increased free fatty acid contents resulted into membrane disruption [35] and cell becomes leaky due to which conductivity values of seed’s leachates were higher in all traditional porous packaging materials (Fig 3C and 3D).

Vigorous seeds have higher activities of α-amylases and reduction in activities of α-amylases are an indication of seed deterioration. In aged non-germinating wheat seeds, no activities of α-amylases have been detected in the scutellum region during imbibition [36]. Maximum total soluble sugars has been measured from the seeds stored in hermetic bags at low moisture contents (8 and 10% moisture contents) that indicate low rate of metabolic activities in dry seeds during storage. Seed deterioration in porous packaging materials resulted into decreased total soluble sugars and similar findings were observed in wheat seeds by Lehner [37]. Higher rate of metabolism and Maillard reaction is the main reason for reduction in total soluble sugars [34]. Reducing sugars are produced in seeds by the hydrolysis of stored reserve at high seed moisture contents [34]. Reducing sugars take part in Maillard reaction by attacking on amino group of protein and nucleic acid/protein complexes and thus have major role in seed deterioration [38].

From the results of this study, it is evident that higher seed moisture is the main culprit for seed deterioration. Seed in traditional storage bags gained moisture from the ambient air of high RH (Fig 1B). Bi-plot of seed germination, electrical conductivity of seed leachates, α-amylases activity, reducing sugars, total soluble sugars, and MDA contents showed that high seed moisture content was the main reason of reduced seed germination, α-amylases activity, total soluble sugars and high electrical conductivity of seed leachates, reducing sugars and MDA content (Fig 6).

## Conclusion

In conclusion, maintaining seed dryness in hermetic Super Bag at 8 and 10% seed moisture contents reduced the deteriorative biochemical and physiological anomalies in okra seed. Moisture contents increased in traditional storage bags which was the main reason of less seed viability, vigor, α-amylases activities, total soluble sugars and increased reducing sugars, EC and MDA contents. Seed storage in hermetic Super Bag is highly dependent upon seed moisture contents and storage at 12 and 14% seed moisture contents is not recommended as it reduced seed viability and vigor.

## Supporting information

S1 File (RAR)
